# Auto-Regression Model-Based Off-Line PID Controller Tuning: An Adaptive Strategy for DC Motor Control

**DOI:** 10.3390/mi13081264

**Published:** 2022-08-06

**Authors:** José A. Niembro-Ceceña, Roberto A. Gómez-Loenzo, Juvenal Rodríguez-Reséndiz, Omar Rodríguez-Abreo, Ákos Odry

**Affiliations:** 1 Facultad de Informática, Universidad Autónoma de Querétaro, Querétaro 76230, Mexico; 2 Facultad de Ingeniería, Universidad Autónoma de Querétaro, Querétaro 76010, Mexico; 3 Industrial Technologies Division, Universidad Politécnica de Querétaro, Carretera Estatal 420, El Marques 76240, Mexico; 4 Department of Mechatronics and Automation, University of Szeged, 6724 Szeged, Hungary

**Keywords:** adaptive control, Brushed DC motor, Proportional-Integral-Derivative control, time series, Auto-Regressive Moving Average model

## Abstract

Brushed (B) and Brushless (BL) DC motors constitute the cornerstone of mechatronic systems regardless their sizes (including miniaturized), in which both position and speed control tasks require the application of sophisticated algorithms. This manuscript addresses the initial step using time series analysis to forecast Back EMF values, thereby enabling the elaboration of real-time adaptive fine-tuning strategies for PID controllers in such a control system design problem. An Auto-Regressive Moving Average (ARMA) model is developed to estimate the DC motor parameter, which evolves in time due to the system’s imperfection (i.e., unpredictable duty cycle) and influences the closed-loop performance. The methodology is executed offline; thus, it highlights the applicability of collected BDC motor measurements in time series analysis. The proposed method updates the PID controller gains based on the Simulink ™ controller tuning toolbox. The contribution of this approach is shown in a comparative study that indicates an opportunity to use time series analysis to forecast DC motor parameters, to re-tune PID controller gains, and to obtain similar performance under the same perturbation conditions. The research demonstrates the practical applicability of the proposed method for fine-tuning/re-tuning controllers in real-time. The results show the inclusion of the time series analysis to recalculate controller gains as an alternative for adaptive control.

## 1. Introduction

Direct current (DC) motors have various applications because they are relatively inexpensive and straightforward to build, especially small versions of them are used in appliances, toys, automobiles, and so on, thanks to their compact and reduced structure [1]. The use of DC motors has several advantages, such as simplicity of operation (by varying the input voltage), relatively low cost, and a wide variety of designs that can adapt to any desired machine design. The presence of parametric uncertainties —variations in the operating environment, presence of noise, and wear of the plant after prolonged and continuous periods of use— is a problem that is faced when operating this type of motor. These parametric uncertainties can end up causing variations in the parameters of the DC motor (in the constants of the motor model) as well as noise in the input and output signal, among other effects that are difficult to predict and that cause inaccuracies in the operation of the control system [2,3]. DC motors work under given specifications depending on the application they are designed for. In some cases, a DC motor may require its shaft to be speed-regulated to a fixed value (within a certain tolerance). There are specific applications for controlling angular speed and positioning of the system as described in [4] that require optimal control development to guarantee performance. For this purpose, the DC motor parameters must be known, i.e., to perform a system identification on the DC motor speed response, which is derived from the interaction of its constituent parts, on a case-by-case basis. This system identification consists, in general, of providing an input signal to the motor and then analyzing the speed response it produces. This reveals the transfer function that describes the input–output relationship. Thus, the transfer function is the base for developing a control system for the DC motor, and the system identification is a way to find a mathematical model for the corresponding dynamic system [5]. DC motors are suitable for mechatronic systems, including those miniaturized. Thus, the miniature robot constitutes a research platform for dynamically re-configurable systems. Path planning or digital image processing algorithms are based on capable data processing. Those tasks are done through a decentralized micro-controller, a high-performance digital signal processor, and an optional re-configurable logical unit (Field Programmable Gate Array), among other characteristics [6].

Modern control and other techniques such as adaptive and artificial intelligence control for changing gains are currently used as alternatives for adaptation mechanisms to system changes in time. In [7], machine learning was used to optimize temperature control for laser instruments. Selecting the attention to specific tasks as an exhibition of cognitive and neural mechanisms was mentioned in [8] for control purposes as an efficient strategy toward adaptive control. Bio-inspired algorithms were presented in [2]; all these meta-heuristic techniques add to continuing with adaptation as a plausible idea for making controllers. Therefore, the authors of this paper propose the use of time series as a novel methodology and find the following:

1. Finding angular speed vs. voltage ratio and a method to forecast it.
2. A strategy to re-tune PID gains off-line by changing angular speed vs. voltage ratio.
3. An option so that the controller can adapt its gains based on current parameters.

The findings are achieved by using a time series analysis to build an Auto-regressive (AR) Integrated (I) Moving Average (MA) model to estimate the changing DC motor parameter, which diverges from its original value in time. The dynamic of a system will change from its initial condition. Although adapting gains has proven to be beneficial, exploring the opportunity to use a novel methodology based on time series would result in additional benefits beyond re-tuning gains as necessary. Time series analysis includes understanding DC motor evolution on continuous operation imposed to a particular disturbance that might follow a regular load pattern.

This research work intends to offer an approach to changing DC motor parameters to update controller gains. It is assumed that the transfer function changes in time, affecting performance. The use of time series analysis helps predict the value of the angular speed vs. voltage ratio to figure out and understand the system’s dynamic. The ARIMA model is used to forecast and these values are used to re-tune the PID controller for comparative analysis. Load accept and load reject scenarios are used to fulfill the following control requirements for testing purposes:

- Overshoot shall be less than 10%.
- Settling time less or equal to 0.25 s.
- No error at steady state (0%).

The remainder of this paper is organized as follows: Section 2 describes relevant foundations and concepts regarding DC motors, nonlinearities, and PID control necessary for this study. Section 3 details the findings derived from the work carried out, including simulation and testing. Finally, Section 4 enunciates the discussion and potential coming work. Section 5 remarks on the conclusions.

## 2. Methods

This section describes the foundations of DC motor transfer function development, identification as a first-order system, a PID controller development, and the analysis of time series and its application to re-tune PID gains offline as an adaptive control alternative.

### 2.1. Theoretical Basis

A DC motor uses magnetic flux, and the current is carried through a conductor, which produces a force on the shaft to generate an angular speed [9]. DC motors can be modeled through the relationship involving electrical and mechanical interactions to develop an electro-mechanical model. This model involves the electrical parameters such as voltage, current, inductance, and resistance, and the mechanical parameters such as friction, torque, and damping ratio, among other features [10]. The model can deduce how these aspects have been associated with each other through the torque-current and voltage constant [11].

- The torque is proportional to the current. This relationship relies on the torque sensitivity constant ($K_{t}$).
- The back electromotive force voltage is proportional to the angular velocity. This proportion depends on the voltage constant ($K_{b}$).

DC motors are exposed to variations (parametric uncertainties) that hinder their operation over time. Strategies such as robust control, optimal control, or offline correction of gains to update controller parameters, among others, are options to meet control requirements. Although these techniques focus on correcting some aspects of the system, sometimes the variations are not well understood or predictable, thus the problem may happen again in the short term. There are few studies to understand how those variations will impact, much less how to anticipate them.

Adaptive and modern control are alternatives for re-tuning the controller gains to current conditions. Several methods for catching the changes in the system can be applied, such as specific algorithms for updating controller parameters, the use of Artificial Intelligence (AI), Fuzzy Logic Controllers (FLC), Neural Networks (NN) for tuning gains, etc. Not all the methods are suitable for all the applications. It is necessary to consider resource consumption and pre-work needed, i.e., training of NNs. In addition, another methodology uses bio-inspired meta-heuristic algorithms that have produced an excellent cost-benefit ratio in terms of resource use contrasted to controller operation [2]. A precise DC motor model is needed for analytical control system design and optimization. Sometimes, reference parameter values of the DC motor detailed in the specification are inadequate. This is the case for cheaper DC motors with relatively large tolerances in the electrical and mechanical parameters. Other identification methods are algebraic and open-loop identification for estimating motor parameters [12], a particular methodology is the identification of the DC motor as a first-order system.

#### 2.1.1. Transfer Function (TF) for a Direct Current (DC) Motor

**Figure 1:**
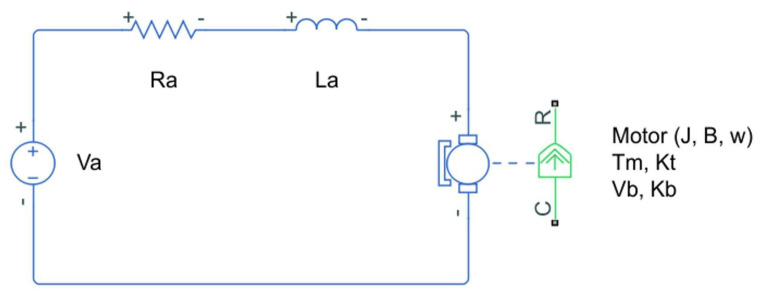
DC motor electro-mechanical schematic; w=ω is the angular speed generated by an input voltage $V_{a}$.

Properly controlled DC motors can achieve precise position, good angular speed regulation, and torque control. Control methods produce the desired response by adjusting gain values that depend on physical parameters; therefore, precisely identifying these parameters becomes relevant. Before any motion, the armature resistance $R_{a}$, the armature inductance $L_{a}$, the back-EMF $K_{b}$, and the torque ratio $K_{t}$ are constant, but once a DC motor is in operation, these values might change due to magnetic effects, among other reasons. In addition, other parameters such as the moment of inertia *J*, change due to the addition or subtractions of mass in the motor shaft [13]. Figure 1 shows a schematic of a DC motor.

The model comprises input voltage $V_{a}$, motor damping *B* attached to the mechanical constituents in the motor, the angular speed ω, and the electrical current *i* (or $I_{a}$) flowing in the circuit.

The governing equation—based on Kirchhoff’s law—for the electrical part is

(1) $$V_{a}=R_{a}i+L_{a}\frac{di}{dt}+V_{b},$$

while, for the mechanical part, the governing equation is

(2) $$J\frac{d\omega }{dt}=T_{m}-B\omega .$$

The torque at shaft $T_{m}$ is generally defined as a combination of different torques such as the cogging torque, the kinetic friction, and the viscous friction (also known as viscous damping force) [12]. It is necessary to express the torque in the shaft as a function of current and the input voltage as a function of angular speed. This results in the following equations:

(3) $$\begin{array}{cc} T_{m} & =K_{t}i, \end{array}$$

(4) $$\begin{array}{cc} V_{b} & =K_{b}\omega . \end{array}$$

Equations (1)–(4) are rewritten as a single system of equations in the Laplace domain as follows:

(5) $$\begin{array}{cc} V_{a}\left(s\right) & =R_{a}I\left(s\right)+L_{a}sI\left(s\right)+K_{b}W\left(s\right), \end{array}$$

(6) $$\begin{array}{cc} JsW(s) & =T_{m}\left(s\right)-BW\left(s\right), \end{array}$$

(7) $$\begin{array}{cc} T_{m}\left(s\right) & =K_{t}I\left(s\right), \end{array}$$

(8) $$\begin{array}{cc} V_{b}\left(s\right) & =K_{b}W\left(s\right). \end{array}$$

By substituting $T_{m}$ from Equation (7) into Equation (6) and substituting $V_{b}$ from Equation (6) into Equation (5), the system can be expressed as the relationship that exists between the angular speed (output—ω) and the input voltage $V_{a}$, that is,

(9) $$\frac{W(s)}{V_{a}\left(s\right)}=\frac{K_{s}}{\frac{L_{a}J}{R_{a}B+K_{b}K_{t}}s^{2}+\frac{L_{a}B}{R_{a}B+K_{b}K_{t}}s+\frac{R_{a}J}{R_{a}B+K_{b}K_{t}}s+1},$$

where $K_{s}$ is defined as

(10) $$K_{s}=\frac{K_{t}}{R_{a}B+K_{b}K_{t}}.$$

The system’s time response is composed of the electrical time constant and the mechanical time constant (intrinsic to each motor). Assuming the electrical time constant is small versus the mechanical time constant, the terms in Equation (9) related to the electrical time constant can be neglected [14]. Equation (9) can be expressed as

(11) $$\frac{W(s)}{V_{a}\left(s\right)}=\frac{K_{s}}{\tau_{s}s+1},$$

where $\tau_{s}$ is defined as

(12) $$\tau_{s}=\frac{R_{a}J}{R_{a}B+K_{b}K_{t}}.$$

**Figure 2:**
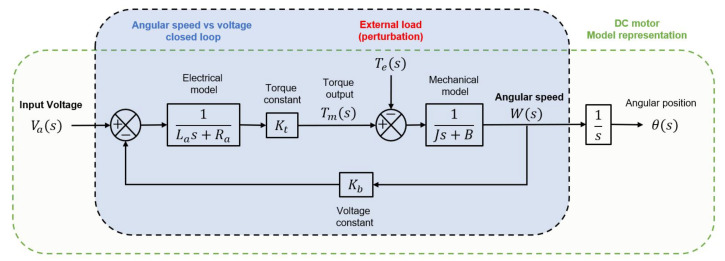
The DC motor block diagram represents the electrical and mechanical components of the device. The electrical component is related to the mechanical component through the torque constant $K_{t}$. The angular speed and voltage input are related through the Back EMF constant $K_{b}$.

The model can be described in the block diagram shown in Figure 2. It is possible to identify each component (the block diagram does not include nonlinearities). The back-EMF and the torque constant play a vital role in the motor operation and the control definition.

#### 2.1.2. DC Motor Identification

Understanding a system before trading it is essential. A system uses modeling and identification and is understood after analysis. Those are a conjugate pair of activities that should be considered in any system. Physical principles in modeling provide a mathematical description with key parameters in a generic form. The resulting model with generic parameters represents a class of models from which a particular element is defined through the identification and estimation of parameters [15].

There are different methods for DC motor model identification:

1. Step and frequency response.
2. State-space-based for modeling/identification.

The challenge in preparing a state-space-oriented control model is that most of the system identification techniques available are for the input–output model. Experiments are therefore proposed to estimate the parameters of an n-order state-space system. It is critical to split the n-order system into n-first order systems as necessary to obtain equivalent discrete-time models for those first-order systems [16]. A typical transfer function of a first-order system with a time constant τ and steady-state gain *K*, assuming no time delay, is given by:

(13) $$G\left(S\right)=\frac{Y(s)}{U(s)}=\frac{K}{\tau s+1}.$$

Equation (13) is equivalent to Equation (11); therefore, both are appropriate representations of a first-order system model used for DC motor identification. Parameter identification is used to obtain an accurate model of a real system. A complete model provides a suitable platform for further developments of the design or control. The online parameter identification schemes are used to estimate system parameters and monitor changes in parameters and characteristics of the system for a diagnosis related to various technology areas. The identification schemes can be used to update the value of the design parameters specified by manufacturers [17]. It is important to remember that electrical dynamics can be neglected because they are faster than the mechanical dynamics in the motor [18]. Control engineers rely on using the manufacturer parameters from the datasheets to sketch an initial approach. In this study, the DC motor used is 36JX30K/38ZY63-1230 from Dongyangcorp. This motor integrates a gearbox that adds torque capacity but reduces speed in a ratio of 50.8 to 1, which is not considered in the modeling. The manufacturer indicated some parameters that are detailed in Table 1.

#### 2.1.3. Controller Approach

Most of the commercial controllers available for industrial processes are based on classic control theories such as Proportional-Integral-Derivative (PID), and they are of closed architecture [19], as mentioned in [20]. In addition, the authors in [20] explained the use of Fuzzy Logic (FL) as trying to emulate the imprecise human reasoning of physical processes into information that is capable of being handled by an embedded system or computer [21]. Fuzzy Logic has been judged over the years because of its ability to face complex problems without the need for models. These controllers (FL-based) are more flexible albeit more complex than PID controllers since they cover a broader range of operating conditions. They can work with internal and external disturbances of different natures. The design of Fuzzy Logic controllers is more accessible than developing a customized model-based controller. In other words, it is possible to modify the structure, rule base, and display it as a human-performed task for controlling. In addition to the previously mentioned, the authors in [20] established that PID, state-space controllers, artificial neural networks, and FL-based controllers are techniques applied to motion control. The PID controller is defined as follows:

(14) $$u\left(t\right)=k_{p}e\left(t\right)+k_{d}\frac{de(t)}{dt}+k_{i}\int e\left(\tau \right)d\tau ,$$

where $k_{p}$ is the proportional gain, $k_{d}$ is the derivative gain, and $k_{i}$ is the integral gain. A significant disadvantage of this algorithm is the computing of the controller gains [21], as mentioned in [20].

The DC motor requires a feedback system to be controlled. When a DC motor includes a feedback system, it can be considered a servo system. Servo systems can have either the position, speed, acceleration outputs, or a combination of these [22]. The PID controllers are programmed based on the original estimation of a TF and cannot be changed during the operation until a software update is loaded if the system allows it. The authors in [22] proposed using an open architecture controller using reconfigurable hardware and a Genetic Algorithm (GA) for an online self-tuning strategy for positioning a linear motion system. The PID algorithm was discretized; then, the gains were calculated using Ziegler–Nichols without needing any model.

In [23], a testing methodology was developed for creating control systems. It helped to discover and implement a mathematical model, estimate different motor model parameters, get familiar with the hardware and software to develop the controller, and use the ATMega328 microprocessor Unit (MCU) as the Central Processing Unit (CPU) device capable of handling the fuzzy logic controller. Using and controlling the motor current besides the motor speed utilizing an FLC is crucial to reaching a more robust controller. This is mentioned in [24]. The authors based this on a review of different proposed control systems. Then, they used FLC with an optimization based on Genetic Algorithms to provide a softer system that can be interpreted as better protecting motor gear-pair, the mechanics, and the feed. The fuzzy logic controller achieved the required behavior by meeting the control requirements. Its robustness was proved by better values from the simulation of the testing scenarios, i.e., the current behaved more smoothly than the PID controller.

### 2.2. Modeling and Controlling the DC Motor through Its Transfer Function (TF)

**Figure 3:**
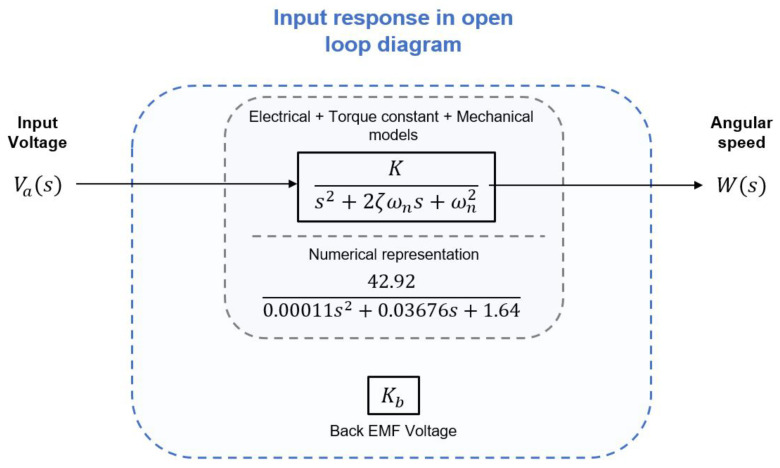
Simplified transfer function in open loop. The Back EMF $K_{b}$ is disconnected from the loop.

The transfer function of the DC motor is deduced from the model shown in Figure 2. It is developed considering the manufacturer’s datasheet. Then, the transfer function is simplified and shown in Figure 3, which determines the angular speed produced by the voltage input. This is also known as the open-loop transfer function that estimates the angular speed vs. voltage ratio.

**Figure 4:**
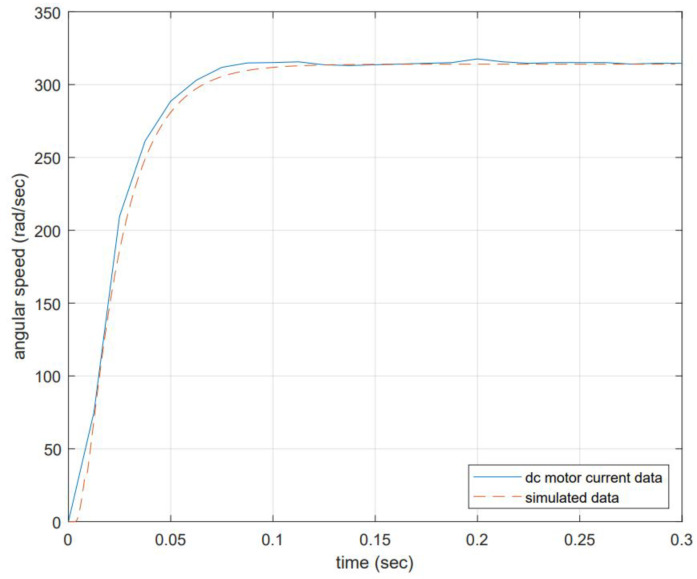
Open-loop system response to a voltage input of 12 volts. The angular speed produced is proportional to the input voltage and is equal to 314.16 rad/s. The dynamic response of the system achieves 63% of the reference angular velocity at 0.025 s, the steady state error at 0% is achieved after 0.15 s.

The DC motor response is compared to the proposed model, and the results are shown in Figure 4. The input voltage is 12 volts at no-load conditions, and the expected angular speed equals 314.16 rad/s.

The model representation is close to the actual data but not exact. The difference might be due to the moment of inertia and viscous friction not matching actual values. Electrical component values might not be as precise as the real values, and nonlinearities are not included (although this might influence much more at low voltages), among others. This testing shows that the simplified transfer function allows an accurate representation which confirms that neglecting some aspects of the complete version is permitted. $K_{s}$ is the inverse of $K_{b}$. This is relevant since this definition and considering the DC motor will change its efficiency in time, then Kb will be estimated in time. Equation (10) can be rewritten as follows:

(15) $$K_{s}=\frac{1}{K_{b}}.$$

#### 2.2.1. Open-Loop Simulation for Gathering Data

**Figure 5:**
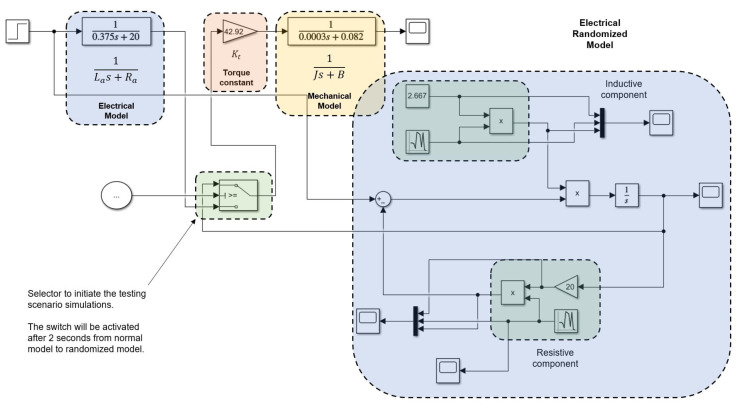
The open-loop simulation produces different Back EMFs considering changes in electrical resistance and electrical inductance values.

**Figure 6:**
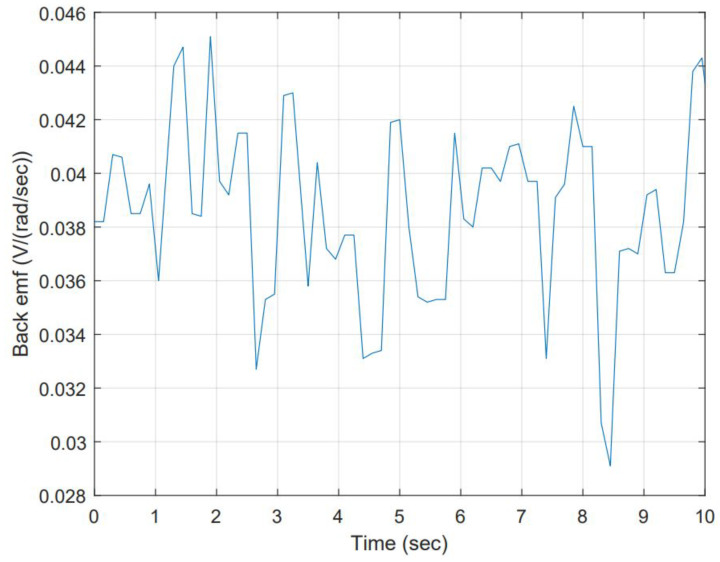
Data collection from simulation for Back EMF. This data collection is presented in time.

The open-loop model helps to simulate scenarios where the electrical resistance and inductance values vary in time by as much as 1% and 0.5%, respectively. This resulted in producing a set of $K_{b}$ values to build a time series, then using it to forecast $K_{b}$ for re-tuning purposes. Simulation is carried out considering a 9 volts step input, seed 0 for electrical component, and seed 1 for inductance component. Figure 5 shows the schematic, and Figure 6 shows the resulting data.

Collecting data can be replicated as often as needed, including changing input steps and percentages of variation and seeds.

#### 2.2.2. PID Controller Design for Closed-Loop Simulation

**Figure 7:**
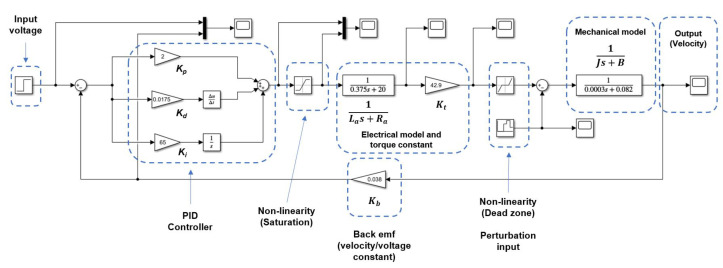
Block diagram representation for the closed-loop system including the PID controller.

**Figure 8:**
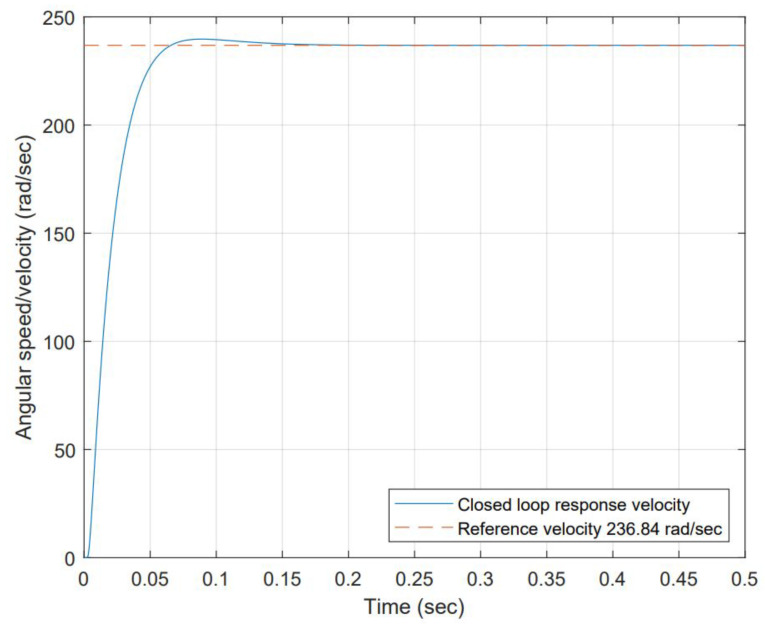
System response to a 9 volts step input in a closed-loop configuration. The system exhibits an under-damped response achieving 63% of reference angular velocity at 0.025 s. This is consistent with experimental open-loop data. The steady estate 0% error is achieved at 0.2 s at approximately 240 rad/s.

PID controllers or their combinations are used to control either BDC or BLDC motors. PI controller controls the angular speed, current, and commutation [25]. PID controller developed for the closed-loop simulation based on the stability analysis that shows the system is stable and controllable (the poles are both negative and on the real axis ($s_{1}=-40.0843$ and $s_{2}=-205.201$). The Routh–Hurwitz matrix confirmed stability (no sign changes in the first column). PID gains are calculated using the auto-tune function in Simulink™ values selected to avoid actuator saturation as follows: $K_{p}=2.0$, $K_{d}=0.0175$ and $K_{i}=65$. Those gains produce an under-damped response, not exceeding 10% of overshoot and they reach settling time in approximately 0.2 s when a step of 9 volts is used as input. The closed-loop block diagram is shown in Figure 7, and its response to 9 volts step input is shown in Figure 8.

### 2.3. The Time Series (TS) Analysis and Stationary Check for the Proposed Model

A time series is a set of observations taken equally in time, and its analysis is concerned with understanding the dependency intrinsic to the data in it. This requires the development of stochastic and dynamic models. A discrete system is one from which observations are taken at equally spaced time intervals [26]. The application of time series and dynamic models is found in areas such as:

- Forecasting a time series’s future values from current and past values.
- The determination of the transfer function of a system is subject to inertia.
- The design of simple control schemes utilizing potential deviations in the system output is compensated by adjusting the input variables.

**Figure 9:**
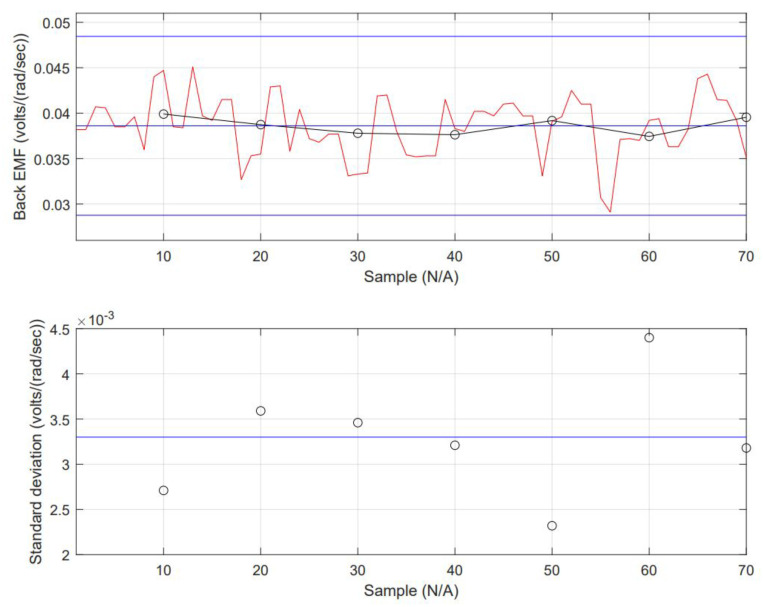
Mean and Standard deviation chart of simulated Back EMF data. The data is displayed in 10 sets of 7 elements each.

The collected data from the simulation can be considered a time series. Verifying the dataset is essential to determine the model that fits better to forecast Back EMF. Stationary criteria are critical to selecting a model, depending on the statistically significant lags, from either the following: Auto-Regressive (AR), Moving Average (MA), including the Integrated (I) aspect (lag reduction), Auto-Regressive Moving Range (ARMA) or Auto-Regressive Integrated Moving Range (ARIMA). Forecasting data is a challenge when it goes far from the last sample. Going back to the stationary check, the data’s nature can be determined. The data’s mean (μ) is constant (this can be interpreted as the mean contrasted to subgroups averages and is not changing drastically). The standard deviation (σ) is also constant. There is no seasonality (not an evident repetitive pattern in time). The inspection of input data is shown in Figure 9. The mean equals 0.0386 and local averages are close. The standard deviation equals 0.00327 with no excessive variabilities in data along the total sampling. All these are shown in Figure 9.

Even though this gives enough evidence of the stationary, there are a couple of additional forms to check it: (1) the global versus local tests and (2) the Augmented Dickey–Fuller (ADF) test. Further details are found in [26].

The stationary requirement has been checked for the dataset; therefore, it is used for extrapolation. The model type can be either the AR or the MA or a combination including the I (ensuring the mean is equal to 0 as a condition to improve fitting). Regression models use past values to predict current values; they are assisted using coefficients inferred from the dataset and the error. The general form has the following equation:

(16) $$\tilde{z}=\phi_{1}{\tilde{x}}_{1}+\phi_{2}{\tilde{x}}_{2}+\cdots +\phi_{p}{\tilde{x}}_{p}+a$$

where ϕ is the *p*th degree polynomial, *a* is the random error term, the $\tilde{x}$ is the independent variable (past values), and the $\tilde{z}$ is the dependent value.

In the Auto-Regressive (AR) model, the current value is expressed as a finite, linear aggregate of previous values and a random constant element $a_{t}$ when the values of a process are at an equally spaced time *t*, t−1, t−2, … by $z_{t}$, $z_{t-1}$, $z_{t-2}$, …In addition, let ${\tilde{z}}_{t}=z_{t}-\mu$ be the series of deviation from μ. It can be defined as follows:

(17) $${\tilde{z}}_{t}=\phi_{1}{\tilde{z}}_{1}+\phi_{2}{\tilde{z}}_{2}+\cdots +\phi_{p}{\tilde{z}}_{p}+a_{t}.$$

In the Moving Average (MA) model, the first consideration is the fact that it is finite. The $\tilde{z}$ is linearly dependent on a finite number of *q* of previous $a_{t}$’s. θ is the *q*th degree polynomial; therefore, the MA model is written as:

(18) $${\tilde{z}}_{t}=a_{t}-\theta_{1}a_{t-1}-\theta_{2}a_{t-2}-\cdots -\theta_{q}a_{t-q}.$$

ϕ and θ are defined as follows:

(19) $$\phi \left(z\right)=1-\phi_{1}z-\ldots -\phi_{p}z^{p}$$

(20) $$\theta \left(z\right)=1-\theta_{1}z-\ldots -\theta_{q}z^{q}$$

So, Equation (17) is known as the AR model of order *p* and Equation (18) is known as the MA process of order *q* [26].

In the AR models, the weights (ψ) are forced to follow an exponential decay form with ϕ as the decay rate. Since the weights are only restricted to the condition $\sum_{i=0}^{\infty }\psi_{i}^{p}<\infty$, it might not be possible to approximate them by an exponential decay pattern. There is a need to increase the order of the AR model to approximate any pattern these weights might exhibit. Adjusting to the exponential decay pattern is sometimes possible by adding a few terms to have a more economical model. In the case of an AR model, disregarding the order is not enough in fulfilling this pattern condition. Instead of increasing its order, it is preferred to add an MA term that will adjust the $\psi_{1}$ while not affecting the rate of the exponential decay pattern for the rest of the weights [27].

A combination of AR(*p*) with MA(*q*) is named Auto-Regressive Moving Average ARMA(p,q) model [26] and it is defined as follows:

(21) $${\tilde{z}}_{t}=\phi_{1}{\tilde{z}}_{1}+\phi_{2}{\tilde{z}}_{2}+\cdots +\phi_{p}{\tilde{z}}_{p}+a_{t}-\theta_{1}a_{t-1}-\theta_{2}a_{t-2}-\cdots -\theta_{q}a_{t-q}$$

**Figure 10:**
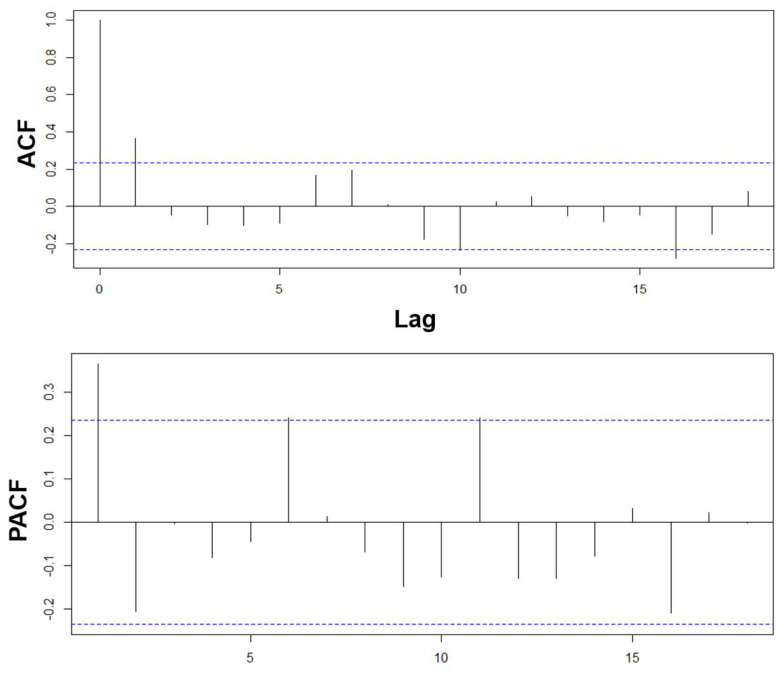
Lag analysis of the time series determines *p*th and *q*th elements depending on statistical significance. The model is AR(1)I(0)MA(1).

The Auto-Correlation Function (ACF) and the Partial Auto-Correlation Function (PACF) are used to determine the number of components the model will include, see Figure 10. The ACF helps more for MA models, while PACF is better for finding the number of elements for AR models. ACF is a good tool for checking the randomness of the data; this is appropriately defined as the Box–Jenkins Auto-Regressive formulation. In MA(*q*), the ACF helps to determine which lags are statistically significant, then define the order of the MA model [28]. PACF can be seen as the correlation between two variables after being adjusted for a common factor affecting them. Considering the formulation proposed by Yule–Walker, which accounts for the formal definition for PACF, for an AR(*p*) process, it is understood that the vector of ${\hat{\phi }}_{kk}$ for any k>p uses the last $\phi_{kk}$ coefficient, called the partial Auto-Correlation of the process at lag *k*, as the cut off after lag *p*. This suggests that the PACF can be used in identifying the order of the AR(*p*) model [27].

**Figure 11:**
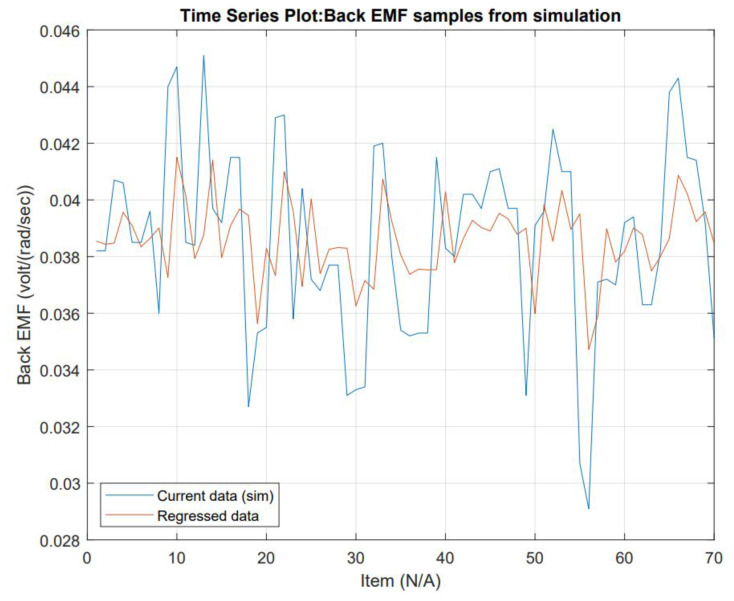
Regressed data displayed with current data to show regression fitting accuracy. Regression was produced using an ARMA model. The current ARMA Model is not fitting the current data; there are some causes for this: first, the data mean is not zero, the proposed model is not adding an integrated (*I*) part, the time series analysis using Ljung–Box concluded the data values are independent.

**Figure 12:**
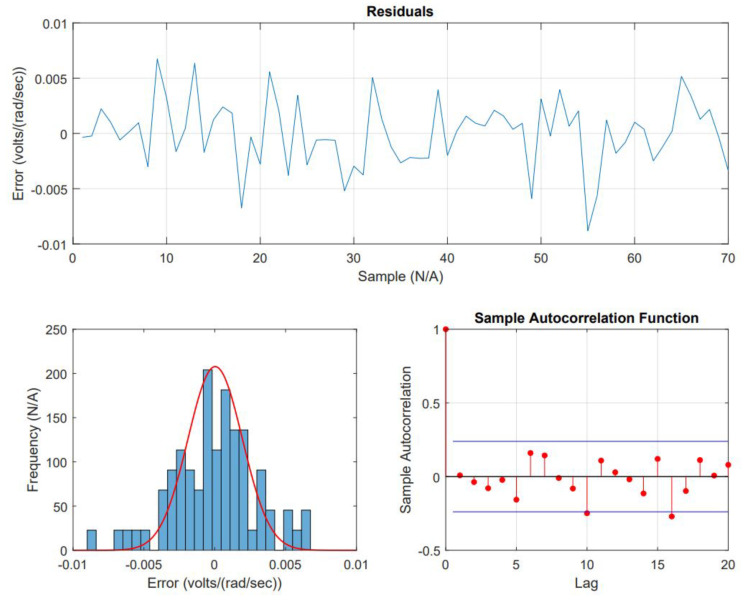
Analysis of residuals between the current data and regressed data. The residuals do not show normal distribution, but a relatively high error.

Coefficients determined, see R™ script used in Appendix A, are AR(1) = 0.0499 with a constant = 0.2289 and MA(1) = 0.3940 and a constant = 0.1997 with a non-zero mean. The fit and wellness of the model are shown in Figure 11 and Figure 12, respectively.

**Figure 13:**
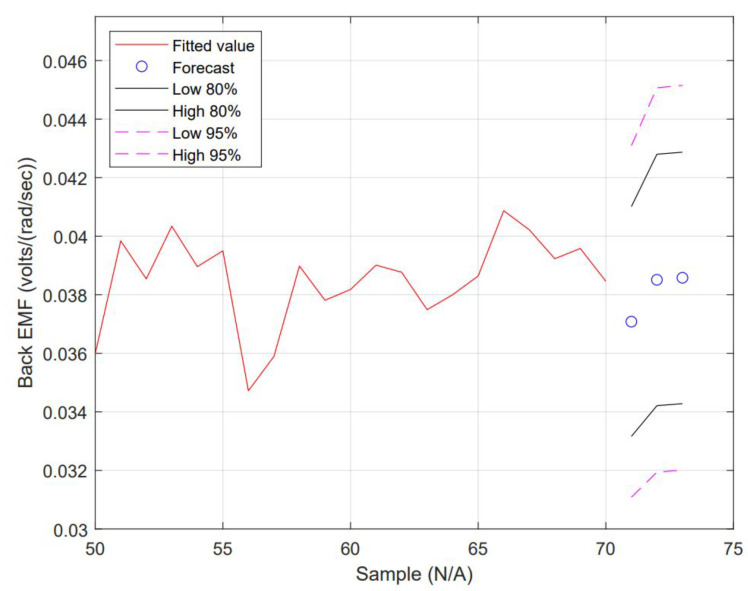
Regression produced with the ARMA model and forecast for the next 3 consecutive values (blue dots shown with CI).

The forecast is shown in Figure 13. All forecast data are detailed in Table 2.

Item 1 from Table 2 is selected for simulation testing to exercise PID gains re-tuning off-line. A detailed description is given in Section 2.4.

### 2.4. Proposed Testing for Simulation

In this test, 3 out of 15 forecast Back EMF ($K_{b}$) values were selected for testing scenarios. Those are: (1) $K_{b}=0.0385$, (2) $K_{b}=0.0342$, (3) $K_{b}=0.045$. The testing simulation uses $T_{e}$, according to Table 3, to add perturbations into the system and verify the PID controller behavior. Proposed $K_{b}$, as predictions, used to re-do the TF and re-tune PID gains from selected scenarios were also used. The input step is set to 9 volts producing an output velocity of approximately 247 rad/s.

**Figure 14:**
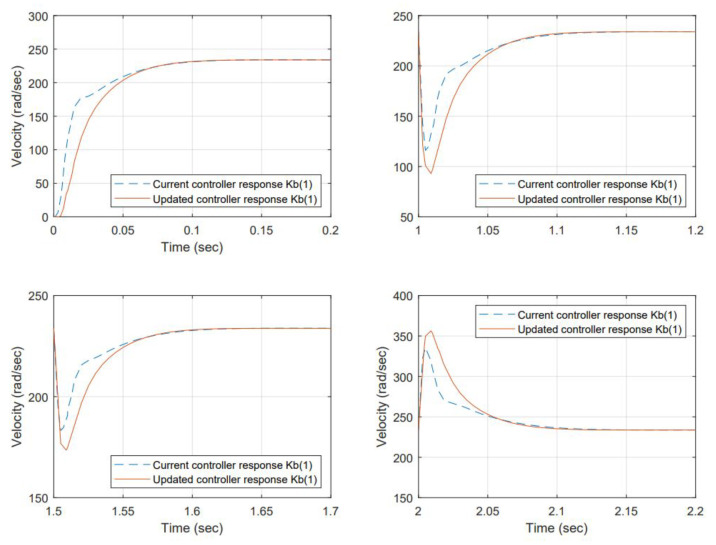
System response to a Back EMF change ($K_{b}=0.0385$)—sample 1. The dynamic response at initial conditions and load accepts and load rejects reaches 63% of the reference at approximately 0.025 sec, the steady-state error is 0% at the stable point after 0.15 s. In terms of performance, the proposed gains have room for improvement to reduce overshoot and increase speed response.

**Figure 15:**
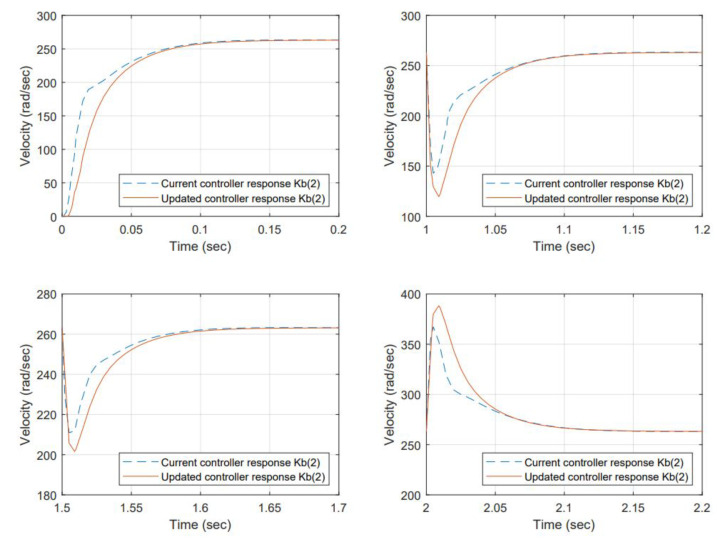
System response to a Back EMF change ($K_{b}=0.0342$)—sample 2. The dynamic response at initial conditions and load accepts and load rejects reaches 63% of the reference approximately at 0.025 s, the steady-state error is 0% at the stable point after 0.15 s. In terms of performance, the proposed gains have room for improvement to reduce overshoot and increase speed response.

**Figure 16:**
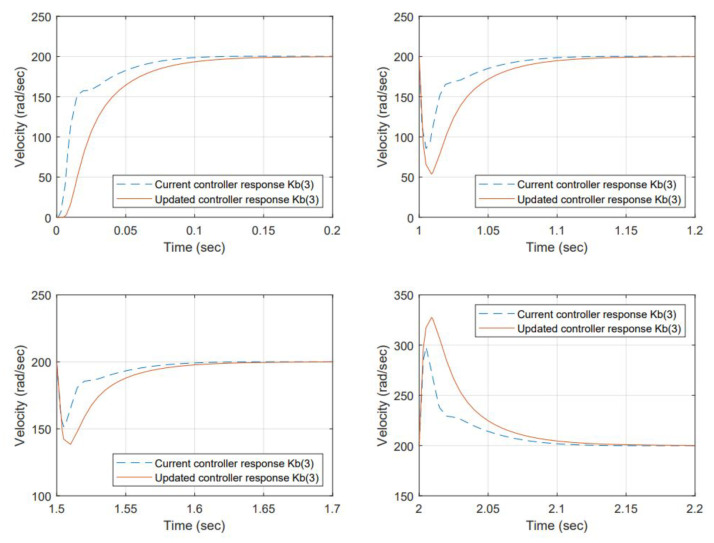
System response to a Back EMF change ($K_{b}=0.045$)—sample 5. The dynamic response at initial conditions and load accepts and load rejects achieves 63% of the reference at approximately 0.025 s, the steady-state error is 0% at the stable point after 0.15 sec. In terms of performance, the proposed gains have room for improvement to reduce overshoot and increase speed response.

Torque values for the perturbation are selected considering the maximum permissible load before a stall condition occurs from the manufacturer’s datasheet. The results for item 1—samples 1, 2, and 5, are shown in Figure 14, Figure 15 and Figure 16 respectively.

With the change of the $K_{b}$, the efficiency has changed, and the resulting angular velocity is different; the current controller is based on regulating voltage for controlling action but not observing any reference angular velocity as a setting. However, comparing the performance back-to-back of the current controller gains is desired, including the change in Back EMF versus the re-tuned (proposed) gains using the auto-tune Simulink ™ function to the new $K_{b}$.

Re-tuned gains improved the damping. The system response is over-damped at a higher level than the original controller at the beginning. However, the load acceptance and load rejection testing scenarios demand more from the system. The gain changes produced a smoother response than that of the original.

## 3. Findings

Highlights of the results are shown below.

### 3.1. In Terms of Control Performance

In this paper, the authors described the development of a PID controller using the classical methodology of inferring the TF by dividing the DC motor components into electrical, mechanical, torque constant, Back EMF constant, and perturbation load, and determining a simplified TF neglecting some aspects of the model. The motor constant was inversely proportional to the Back EMF $K_{b}$ constant (which resulted in the simulation of the time series used in the proposed adaptive strategy). The values for the model were taken from the manufacturer’s datasheet. With these in mind, an open-loop stimulus is executed into the physical system to know the DC motor response to an input of 12 volts that produced an angular velocity of 314.16 rad/s. This response was compared with the proposed system simulation and it matched with minor differences associated with the moment of inertia and friction not precisely modeled.

The model, in general terms, is considered suitable to create a closed-loop system, including a PID controller in which the gains were auto-tuned using the Simulink ™ function to get a performance of an under-damped system not exceeding 10% of overshoot and settling time less than 0.2 s. In case of not meeting any control requirements, the gains would be updated to get the desired results while ensuring the actuator is not overloaded. Two nonlinearities are included in the closed-loop model: saturation and dead zone. It is believed that hysteresis can be analyzed after reviewing the DC motor behavior functioning for a period. It was then measured to see if changes occurred. (This paper is focusing on simulation for now. Therefore, this was not accounted for in the model.) The friction is similar to hysteresis because it is also needed to carry out experiments with no load and loads to determine the friction in static and dynamic conditions. Both will be included later to increase the modeling robustness. The proposed system is considered suitable for simulation.

### 3.2. In Terms of Time Series, Assuming a Shortened Time Scenario to Collect Data

The first aspect to consider is that the time series is simulated by creating a random environment and producing changes in the inductive and resistive components of the DC motor. The randomization consisted of using a mean equal to 1 for both cases and 0.5% up and down and 1% up and down variations with seeds 1 and 0, respectively. This simulation was carried out in an open loop, and a new set of $K_{b}$ was built along 15 s of simulation continuously produced every 0.001 sec. However, the data gathered were processed to get samples every 0.15 s, with a total of 70 items and used to build the time series. The analysis determined the time series as stationary and was then checked using ADF. ACF and PACF plots were used to find lags, resulting in lag 1 for both the Auto-Regressive (AR) and Moving Average (MA). The forecast is built considering the previous iteration and no more. The regression part of the model showed some degree of error while attempting to estimate data. The residual analysis pointed to the lack of exactness in the proposed ARMA model, but also a factor was that the resulting model did not account for 0 mean nor included the model’s integrated (*I*) aspect. Room for improvement, which is being reviewed for improving fitting.

The model was suitable for forecasting at least three rounds of successive future values. The second was selected, including the error band, which made the forecasting much more interesting because this provides power in terms of adaptation. The confidence interval (CI) values are included to expand the scenario for re-tuning the gains. The CI is set to 20% and 5%, respectively. This can be adjusted as needed.

### 3.3. In Terms of an Auto-Regressive Moving Average Model

In terms of the ARMA model, it is worth mentioning that the Integrated (*I*) part is not considered. This does remove lag one from the sample, then centers the data to a 0 mean, a necessary condition for using the *I* component when a time series is not stationary. This component was tested, and the regression behaved similarly to ARMA. More investigation on this is foreseen.

For the adaptive strategy, an increasing time series accuracy is essential for better regression. In this case, real data exploration is advised to improve this technique.

### 3.4. In Terms of Comparing Performance between the Controllers

The controller performance for selected scenarios is compared back-to-back. The change in $K_{b}$ produced a difference in the output angular velocity. The gains were re-tuned using a toolbox included in Simulink ™ offline; therefore, in that regard, the criteria used were to over-damp the system and produce a smoother response, increasing settling time at no cost. However, the difference was insignificant because the response was fast enough for all cases. The proposed gains were less tolerant to load accept and load reject testing scenarios. In this matter, it is necessary to work on finding a different methodology to improve this condition. For the initial state, the re-tuned controllers were behaving better.

Finally, it is worth mentioning that using the ARMA model to re-tune PID gains is suitable for developing robust, more tolerant, and intelligent control. In addition, forecasting DC motor parameters can be beneficial in predicting other operational risks.

## 4. Discussion

After reviewing the results, the following outcomes are presented.

### 4.1. Conceptual Analysis

During this experiment, it was found that time series can be used to produce Auto-Regressive Moving Average models to assist in re-tuning gains of existing PID controllers using tools for that purpose off-line. The classical methodology for building a PID controller is exposed here among the use of time series analysis to develop an ARMA model to forecast $K_{b}$ values in a fast-degrading scenario for a DC motor operation considering up and down conditions. Accurate data would be better to understand DC motor conditioning after its operation; however, the idea expressed here is related to DC motor changing in time for an extended period. The sampling was shortened to manage raw data but was helpful in this development. The scenarios simulated were good for building the time series, and a strategy to adapt PID gains offline is presented. This has room for improvement, and some ideas can be explored soon in this area.

### 4.2. Using Physical Means for Testing and Developing Real Time Series

Testing with a real device is better since it adds uncertainty. For years, adapting any controller to the changes the system might experience has been a topic of study, ending up in building exceptional controllers that use computational resources but are hard to implement in industrial processes. Exploring a simple method for creating an adaption strategy in a PID controller might be attractive if it does not consume a lot of resources while embedded.

### 4.3. Opportunities for Coming Phases

The next step is to implement these methods online in a real system as embedded software. It is necessary to work separately in implementing the time series algorithms in the micro-controller and do all the calculations for creating the model, either AR or MA or a combination, including the I portion if needed, then think about the online strategy for processing the auto-tune code and technology for a successful implementation. It is recommended to test additional scenarios, besides the step input, such as ramp and sinusoidal inputs, and to include load accepts and load rejects on such inputs for effectiveness review of the proposed algorithm.

## 5. Conclusions

The authors of this paper believe that the proposed technique adds to adaptive control methodologies in the sense of including time series analysis to forecast DC motor parameters and then improve the controller performance in the context of its continuous operation, thinking about the likely changes it will experience due to duty cycle. This is undoubtedly unpredictable; therefore, ARIMA models can aid in understanding these possible system changes. For now, the potential to expand this to other disciplines, such as predictive and intelligent control, can help re-tune gains much more if this is implemented online. For now, the offline experimentation provides an insight into the value of continuing this path. The initial conclusion is that although there is room for improvement, the use of the model seems promising.

## Figures and Tables

**Table 1:** 36JX30K/38ZY63-1230 DC motor parameter value.

	Parameter	Value	Unit
	Armature resistance ($R_{a}$) *	20.0 +/− 10%	Ω
	Armature Inductance ($L_{a}$) *	0.375 +/− 10%	H
Electrical	Rated current ($I_{a}$)	0.65	A
	Stall current ($I_{s}$)	2.4	A
	Output power (*W*)	3.8	W
	Rated voltage ($V_{a}$)	12	V
	Torque constant ($K_{t}$) **	42.92	mN m/A
	Motor inertia (*J*) *	0.0003	$\mathrm{Kg}\;m^{2}$
	Viscous Damping (*B*) *	0.082	mN m s/rad
Mechanical	Back EMF ($K_{b}$)	0.0382	V/(rad/s)
	Rated torque ($T_{m}$)	15.0	mN m
	Rated speed (ω)	261.8	rad/s
	Stall torque ($T_{s}$)	90.0	mN m

* Measured or calculated item. ** At rated conditions.

**Table 2:** Forecast dataset that includes an 80% confidence interval (CI) and 95% CI, both Low and High.

Item	Forecast	Lo 80	Hi 80	Lo 95	Hi 95
0	0.03708248	0.03315761	0.04100735	0.03107991	0.04308505
1	0.03850688	0.03421273	0.04280103	0.03193954	0.04507421
2	0.03857796	0.03428293	0.04287299	0.03200928	0.04514664
–>	Sample 1	Sample 2	Sample 3	Sample 4	Sample 5

**Table 3:** Proposed loads for simulation and testing.

Item	Load	Units	Timing
1	0	mN m	0.5 s
2	0	mN m	0.5 s
3	15.75	mN m	0.5 s
4	22.5	mN m	0.5 s
5	8.75	mN m	0.5 s

## Data Availability

All data presented in this study are available upon request from the corresponding authors.

## Abbreviations

The following abbreviations are used in this manuscript:

ACF	Auto-Correlation Function
ADF	Augmented Dickey–Fuller’s
AI	Artificial Intelligence
AR	Auto-Regressive
ARIMA	Auto-Regressive Integrated Moving Range
ARMA	Auto-Regressive Moving Range
BDC	Brushed Direct Current
BLDC	Brushless Direct Current
CPU	Central Processing Unit
DC	Direct Current
FL	Fuzzy Logic
FLC	Fuzzy Logic Controllers
I	Integrated
MA	Moving Average
MCU	Microprocessor Unit
NN	Neural Networks
PACF	Partial Auto-Correlation Function
PI	Proportional-Integral
PID	Proportional-Integral-Derivative
TF	Transfer Function
TS	Time Series

## Appendix A. Code in R™ for Analyzing the Time Series Data

 # Libraries needed for processing data

 library(tseries)

 library(forecast)

 # Reading csv data and fitting it into a time series in R

 ar.ts <- read.csv("emf_data.csv", header = TRUE, sep = ",")

 AR.ts <- ts(ar.ts, start = c(0,01), frequency = 1)

 # Time series plot to show data for analysis.

 # Check for mean, stddev and seasonality.

 plot(AR.ts)

 # Dickey-Fuller test

 adf.test(AR.ts)

 # Auto-correlation function and Partial Auto-correlation function plots

 acf(AR.ts)

 pacf(AR.ts)

 # Analyzing data from Back EMF sample data set

 # This function gives a complete insight of the data set as time series.

 forecast::tsdisplay(AR.ts)

 # Creating either AR and/or (I)MA or combination

 AR1I0MA1 = forecast::Arima(AR.ts, c(1, 0, 1))

 summary(AR1I0MA1)

 # Plotting original data contrasted to models developed

 plot(AR.ts)

 lines(fitted(AR1I0MA1), col = ’red’)

 # Checking wellness of models created

 forecast::checkresiduals(AR1I0MA1)

 # Predicting values for Back EMF in the next 3 cycles

 PR_mdl_AR1I0MA1 = forecast::forecast(AR1I0MA1, h = 3)

 plot(PR_mdl_AR1I0MA1, col = ’red’) # Plotting the forecast

 PR_mdl_AR1I0MA1 # Displaying the 3 forecast values
